# Complete mitochondrial genome of *Echinophylliaaspera* (Scleractinia, Lobophylliidae): Mitogenome characterization and phylogenetic positioning

**DOI:** 10.3897/zookeys.793.28977

**Published:** 2018-10-29

**Authors:** Wentao Niu, Shuangen Yu, Peng Tian, Jiaguang Xiao

**Affiliations:** 1 Laboratory of Marine Biology and Ecology, Third Institute of Oceanography, State Oceanic Administration, Xiamen, China Laboratory of Marine Biology and Ecology, Third Institute of Oceanography, State Oceanic Administration Xiamen China

**Keywords:** Daya Bay, gene order, next-generation sequence, phylogeny

## Abstract

Lack of mitochondrial genome data of Scleractinia is hampering progress across genetic, systematic, phylogenetic, and evolutionary studies concerning this taxon. Therefore, in this study, the complete mitogenome sequence of the stony coral *Echinophylliaaspera* (Ellis & Solander, 1786), has been decoded for the first time by next generation sequencing and genome assembly. The assembled mitogenome is 17,697 bp in length, containing 13 protein coding genes (PCGs), two transfer RNAs and two ribosomal RNAs. It has the same gene content and gene arrangement as in other Scleractinia. All genes are encoded on the same strand. Most of the PCGs use ATG as the start codon except for ND2, which uses ATT as the start codon. The A+T content of the mitochondrial genome is 65.92% (25.35% A, 40.57% T, 20.65% G, and 13.43% for C). Bayesian and maximum likelihood phylogenetic analysis have been performed using PCGs, and the result shows that *E.aspera* clustered closely with *Sclerophylliamaxima* (Sheppard & Salm, 1988), both of which belong to Lobophylliidae, when compared with species belonging to Merulinidae and other scleractinian taxa used as outgroups. The complete mitogenome of *E.aspera* provides essential and important DNA molecular data for further phylogenetic and evolutionary analyses of corals.

## Introduction

Reef-building coral species of the order Scleractinia play an important role in shallow tropical seas by providing an environmental base for the ecosystem (Fukami et al. 2000). These coral species have been traditionally described using morphological character traits of skeletons as demonstrated in various taxonomic revisions published in the last century (Dinesen 1980; Hoeksema 1989; Wallace 1999). Traditional morphology-based systematics does not reflect all the evolutionary relationships of Scleractinia, which therefore forms a problematic group for taxonomy. Environment-induced phenotypic variation, morphological plasticity, evolutionary convergence of skeletal characters, intraspecific variation caused by different genotypes, and genetic mixing via introgression cause intraspecific and interspecific variability to overlap (Todd 2008; Combosch and Vollmer 2015; Richards and Hobbs 2015). Molecular data have therefore become increasingly important in recent years to overcome the limitations of morphological analyses among scleractinians (e.g. Benzoni et al. 2011, 2012a, 2014; Gittenberger et al. 2011; Huang et al. 2011, 2014a, 2014b; Budd et al. 2012; Arrigoni et al. 2014a, 2017; Kitano et al. 2014; Schmidt-Roach et al. 2014; Terraneo et al. 2016a, 2017). In particular, the family Lobophylliidae has received much attention recently with regard to its phylogeny (Arrigoni et al. 2014b, 2015, 2016a, 2016b, 2018; Huang et al. 2016).

The unique characters of mitochondrial genome DNA (mitogenome), which include small size, fast evolutionary rate, simple structure, maternal inheritance, and high informational content, suggest that the constituting loci could be powerful markers for resolving ancient phylogenetic relationships (Boore 1999; Sun et al. 2003; Geng et al. 2016). This has also been applied for a number of scleractinian taxa (e.g. Fukami and Knowlton 2005; Flot and Tillier 2007; Wang et al. 2013; Arrigoni et al. 2016c; Capel et al. 2016; Niu et al. 2016; Terraneo et al. 2016b, 2016c). In recent years, next-generation sequencing (NGS), combined with bioinformatic annotation, is becoming increasingly common for recovering animal mitogenome sequences and allows a rapid amplification-free sequencing (Jex et al. 2010). However, the complete mitochondrial genomes of stony corals that we can find in NCBI (National Center for Biotechnology Information) are less than 80 species.

*Echinophylliaaspera* (Ellis & Solander, 1786), commonly known as the chalice coral, is a stony coral species with large polyps in the scleractinian family Lobophylliidae. It is native to the western and central Indo-Pacific (Veron 2000). In this study, we sequenced the complete mitogenome sequence of *E.aspera* for the first time using NGS and analyzed its structure. It is the second lobophylliid species to be examined for its mitogenome after *Sclerophylliamaxima* (Sheppard & Salm, 1988) (Arrigoni et al. 2015, 2016c). Furthermore, we conducted phylogenetic analyses based on the mitochondrial sequence data of this species and 10 other scleractinians with the purpose of investigating the phylogenetic position of *E.aspera*. The mitogenome information reported in this article will facilitate further investigations of evolutionary and phylogenetic relationships of stony corals.

## Materials and methods

### Sample collection and DNA extraction

**Figure 1.**
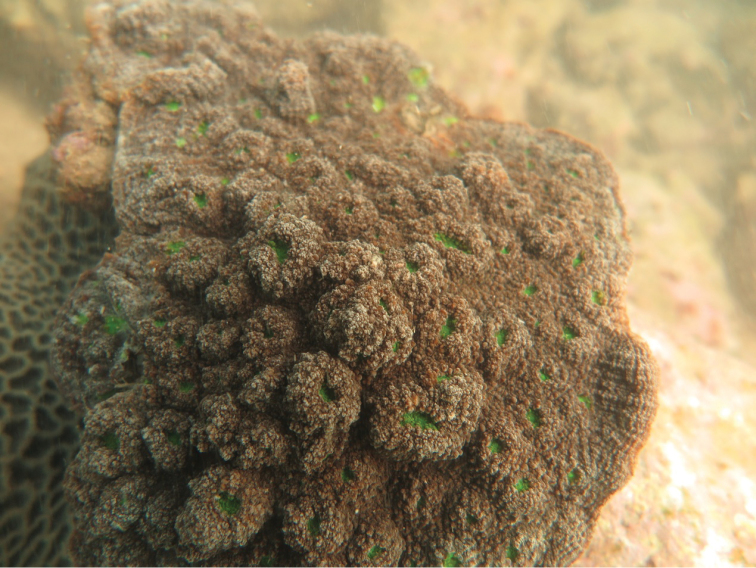
Example of *Echinophylliaaspera* used in the present study.

Samples (voucher no. DYW15) of *Echinophylliaaspera* (Figure 1) were collected from Daya Bay in Guangdong, China. Specimens were identified based on skeletal morphology after detailed observation of corallite features using a dissecting microscope. The number of septa, the number of denticles, the calice, and the dimension were analyzed with reference to taxonomic descriptions (Veron 2000; Arrigoni et al. 2016b). Total genomic DNA was extracted using the DNeasy tissue Kit (Qiagen China, Shanghai) and kept at 4°C for subsequent use.

### Genome sequencing and analyses

We used next generation sequencing to perform low-coverage whole-genome sequencing according to the protocol (Niu et al. 2016). PCR products were subjected to agarose gel, Nanodrop 2000 (Thermo Scientific, USA) and Qubit 2.0 Fluorometer (Life technologies, USA) to confirm its purity and concentration. A total of 2µg double strand DNA (dsDNA) passed the quality control steps were sheared to ~550bp by M220 focused-ultrasonicator (Covaris, Woburn, MA, USA). Fragmented DNA was tested for size distribution by using the Agilent Bioanalyzer 2100 (Agilent Technologies, Santa Clara, CA, USA) and library for Miseq was generated by TruSeq DNA PCR-free LT sample preparation kit (Illumina, San Diego, CA, USA) according to manufacturer’s instructions. Final library concentration was determined by real-time quantitative PCR with Illumina adapter-specific primers provided by KAPA library quantification kit (KAPA Biosystems, Wilmington, MA, USA). About 0.05% raw reads (3,017 out of 6,340,606) were *de novo* assembled by using commercial software (Geneious V9, Auckland, New Zealand) to produce a single, circular form of complete mitogenome with about an average 38 × coverage.

### Mitogenome annotation and analyses

The assembled consensus sequence was further annotated and analyzed. Preliminary annotation using DOGMA (Wyman et al. 2004) and MITOS (Bernt et al. 2013) webserver provided overall information on mitogenome. Protein-coding genes and rRNA genes were annotated by alignments of homologous genes of other reported mitogenome of Scleractinia. Blast searches in the National Center for Biotechnology Information also helped to identify and annotate the PCGs and rRNA genes. Transfer RNA genes were identified by comparing the results predicted by ARWEN based on cloverleaf secondary structure information (Laslett and Canback 2008). Nucleotide frequencies and codon usage were determined by MEGA7 software (Kumar et al. 2016).

### Phylogenetic analyses

To validate the phylogenetic position of *E.aspera* within the Scleractinia, the complete mitogenome sequences of an additional ten representative scleractinian species (Table 1) were incorporated together with the presently obtained *E.aspera* mitogenome sequence for phylogenetic analysis. The phylogenetic trees were built using two approaches including maximum-likelihood (ML) analysis by PAUP* 4.0 (Swofford 2002) and a partitioned Bayesian inference (BI) analysis by Mrbayes 3.12 (Huelsenbeck and Ronquist 2001) based on 13 PCGs binding sequence. The substitution model selection was conducted by a comparison of Akaike Information Criterion (AIC) scores with jModelTest 2 (Darriba et al. 2012). The GTR+I+G model was chosen as the best-fitting model for ML analyses and the node reliability was estimated after 1000 bootstrap replicates. For the Bayesian procedure, four Markov chains were run for 1,000,000 generations by sampling the trees every 1000 generations. After the first 2500 trees (25%) were discarded as burn-in, the 50% majority rule consensus tree and the Bayesian posterior probabilities (BPP) were estimated using the remaining 7500 sampled trees. *Madreporaoculata* Linnaeus, 1758, belonging to Oculinidae was used as outgroup for tree rooting.

**Table 1.** Representative Scleractinia species included in this study for comparison.

Species	Family	Distribution	Length (bp)	GenBank accession number
* Echinophyllia aspera *	Lobophylliidae	Indo-Pacific	17,697	MG792550
* Sclerophyllia maxima *	Lobophylliidae	Indo-Pacific	18,168	FO904931
* Platygyra carnosa *	Merulinidae	Indo-Pacific	16,463	NC_020049
* Favites abdita *	Merulinidae	Indo-Pacific	17,825	NC_035879
* Favites pentagona *	Merulinidae	Indo-Pacific	18,006	NC_034916
* Orbicella faveolata *	Merulinidae	West Atlantic	16,138	AP008978
* Orbicella franksi *	Merulinidae	West Atlantic	16,138	AP008975
* Orbicella annularis *	Merulinidae	West Atlantic	16,138	AP008974
* Mussa angulosa *	Mussidae	West Atlantic	17,245	NC_008163
* Colpophyllia natans *	Mussidae	West Atlantic	16,906	NC_008162
* Madrepora oculata *	Oculinidae	West Atlantic	15,841	NC_018364

## Results and discussion

### Mitochondrial genome organization

**Figure 2.**
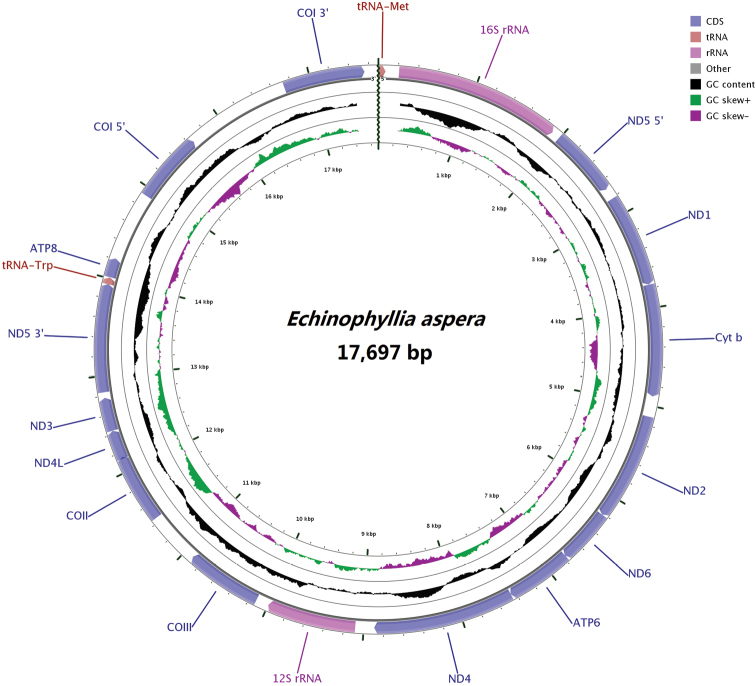
The mitochondrial genome of *Echinophylliaaspera*. Gene order and positions are shown; all the genes are encoded on H-strand. COI, COII, COIII refer to the cytochrome oxidase subunits, Cyt *b* refers to cytochrome b, ND1-ND6 refer to NADH dehydrogenase components.

**Figure 3.**
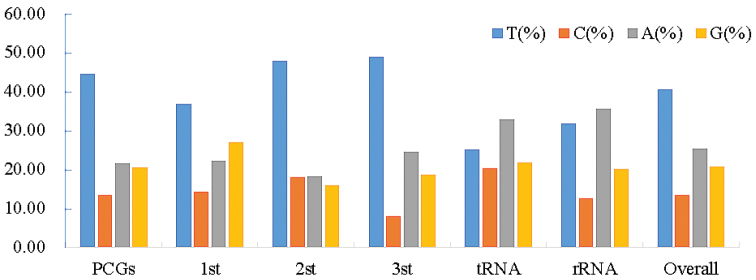
Codon usage bias in different regions of mitochondrial genome of *Echinophylliaaspera*.

The complete mitogenome of *E.aspera* was 17,697 bp in size (GenBank accession number: MG792550) including unique 13 protein-coding genes (PCGs), two transfer RNA genes (tRNA-Met, tRNA-Trp) and two ribosomal RNA genes (Figure 2, Table 2). Its overall base composition was 25.35% for A, 13.43% for C, 20.65% for G and 40.57% for T, and showed a high A+T content with mean overall value of 65.92% (Figure 3, Table 3). All PCGs, tRNA and rRNA genes were encoded on H-strand. The base C was at the lowest level in different regions of the mitogenome (Figure 3). The mitochondrial genome of *E.aspera* provided no peculiar structure; its gene identity, number and order were identical to most of the scleractinian coral mitogenomes already published (Wang et al. 2013).

**Table 2.** Organization of the mitochondrial genome of *Echinophylliaaspera*.

Gene	Position		Length (bp)	Anticodon	Codon		Intergenic nucleotides*	Strand
	From	To			Start	Stop		
tRNA^Met^	1	72	72	UAC			140	H
16S rRNA	210	1905	1696				137	H
ND5 5’	1991	2701	711		ATG		85	H
ND1	2813	3760	948		ATG	TAG	111	H
Cyt *b*	3763	4902	1140		ATG	TAA	2	H
ND2	5111	6214	1104		ATT	TAA	208	H
ND6	6216	6776	561		ATG	TAA	1	H
ATP6	6776	7453	678		ATG	TAA	-1	H
ND4	7453	8892	1440		ATG	TAG	-1	H
12S rRNA	9085	9996	912				192	H
COIII	10127	10906	780		ATG	TAG	130	H
COII	11448	12155	708		ATG	TAA	541	H
ND4L	12137	12436	300		ATG	TAA	-19	H
ND3	12439	12780	342		ATG	TAA	2	H
ND5 3’	12838	13941	1104			TAG	57	H
tRNA^Trp^	13940	14010	71	ACU			-2	H
ATP8	14014	14211	198		ATG	TAA	3	H
COI 5’	14920	15650	731		ATG		708	H
COI 3’	16726	17556	831			TAG	1075	H

Notes: * Data are number of nucleotides between the given gene and its previous gene, negative numbers indicate overlapping nucleotides.

**Table 3.** Nucleotide composition in different regions of mitochondrial genome of *Echinophylliaaspera*.

Gene/Region	T(%)	C(%)	A(%)	G(%)	A+T(%)	Size (bp)
ND5	46.12	12.51	21.60	19.78	67.72	1815
ND1	43.88	13.50	20.57	22.05	64.45	948
Cyt *b*	46.05	13.51	20.88	19.56	66.93	1140
ND2	46.74	13.04	20.29	19.93	67.03	1104
ND6	47.06	13.19	20.86	18.89	67.92	561
ATP6	46.46	14.01	20.65	18.88	67.11	678
ND4	44.65	14.24	20.00	21.11	64.65	1440
COIII	41.40	15.60	20.60	22.30	62.00	780
COII	39.41	12.57	25.71	22.32	65.12	708
ND4L	43.67	10.67	27.33	18.33	71.00	300
ND3	49.71	9.06	18.71	22.51	68.42	342
ATP8	43.43	10.60	33.33	12.63	76.76	198
COI	41.42	14.53	22.60	21.45	64.02	1562
PCGs	44.50	13.40	21.60	20.50	66.10	11576
1^st^	36.70	14.20	22.10	27.00	58.80	3859
2^st^	47.90	18.00	18.20	15.90	66.10	3859
3^st^	48.90	8.00	24.50	18.60	73.40	3858
tRNA	25.17	20.28	32.87	21.68	58.04	143
rRNA	31.75	12.65	35.43	20.17	67.18	2608
Overall	40.57	13.43	25.35	20.65	65.92	17697

### Protein-coding genes

**Figure 4.**
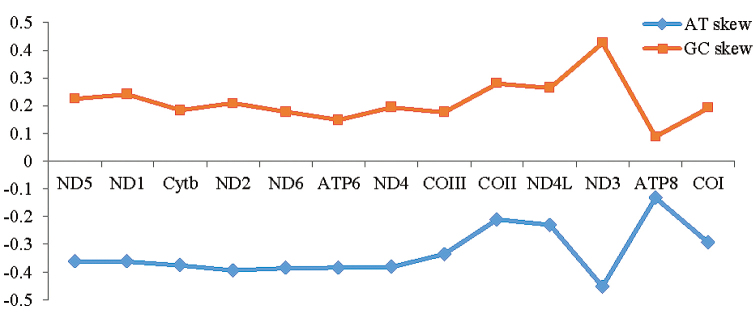
The PCGs’ AT-skew and GC-skew of mitochondrial genome of *Echinophylliaaspera*.

**Figure 5.**
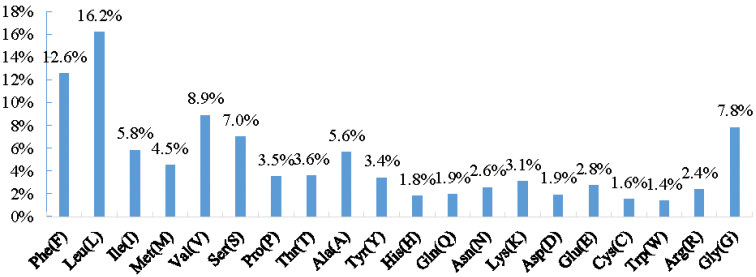
The PCG-codons use frequency of mitochondrial genome of *Echinophylliaaspera*.

The PCGs was 11,576 bp in size, and its base composition was 21.6% for A, 13.4% for C, 20.5% for G and 44.5% for T. The ND5 had a 10,136 bp intron insertion, and COI had a 1,075bp intron insertion. According to Lin et al. (2014), the ND5 intron of *E.aspera* was the canonical scleractinian organization (Type SII), ten proteincoding genes and rns are contained in the ND5 intron. According to Fukami et al. (2007), the group I intron in COI of *E.aspera* was the canonical Type 2, with one deletion of T at position 77. All of the PCGs used ATG as the start codon except for ND2, which used ATT as the start codon. Five of the 13 PCGs were inferred to terminate with TAG (ND1, ND4, ND5, COI and COIII), 8 PCGs with TAA (Cyt *b*, ATP6, ND2, ND4L, ND3, ND6, COII and ATP8). Among 13 PCGs, the longest one was ND5 gene (1,815 bp), whereas the shortest was ATP8 gene (198 bp). There were 1 bp overlapping nucleotides between ND6 and ATP6, 1 bp overlapping nucleotides between ATP6 and ND4, 2 bp overlapping nucleotides between tRNA-Trp and ND5 5’, 19 bp overlapping nucleotides between ND4L and COII, and the number of non-coding nucleotides between different genes varied from 1 to 1075 bp (Table 2). Nucleotide asymmetric research can be measured through the AT-skew and GC-skew method, the calculation formula was: AT skew = (A-T)/(A+T), GC skew = (G-C)/(G+C). According to the results (Figure 4), the PCGs showed stronger AT-skew and GC-skew, the absolute value of AT-skew was greater than GC-skew. Among 3858 codons for 20 amino acids, codons use frequency was higher in L, F, V, I and G, accounted for 53.2% of all amino acids. Nonpolar amino acid (G, A, V, L, I, M, F, Y, W) accounted for 66.2% which was the maximum, followed by polar amino acid (S, P, T, C, N, Q) accounted for 20.2%, the polarity charged amino acids (K, H, R, D, E) accounted for 13.6% which was minimum (Figure 5).

### Ribosomal and transfer RNA genes

**Figure 6.**
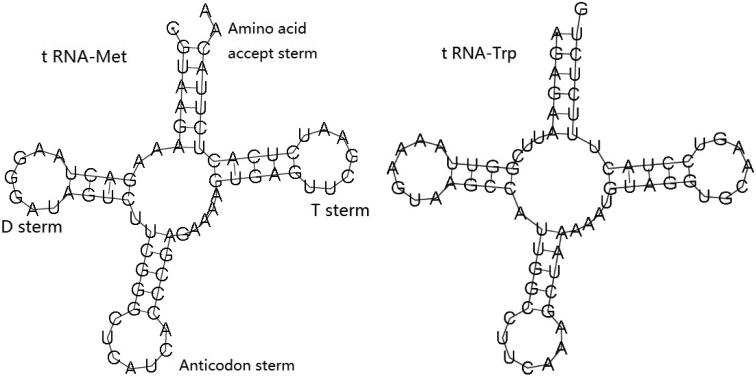
Putative secondary structures of two tRNA of *Echinophylliaaspera*.

The genes encoding the small and large ribosomal RNA subunits (12S rRNA and 16S rRNA) were identified in *E.aspera*, which were 912 bp and 1,696 bp in length, respectively. The total ribosomal RNA was 2,608 bp in size, and its base composition was 35.43% for A, 12.65% for C, 20.17% for G and 31.75% for T. The two transfer RNAs were 72 bp for tRNA-Met and 71 bp for tRNA-Trp in length respectively. They can be folded into the typical cloverleaf structure, the typical cloverleaf structure contained amino acid accept stem, TψC stem, anticodon stem, and DHU stem (Figure 6).

### Phylogenetic analyses

**Figure 7.**
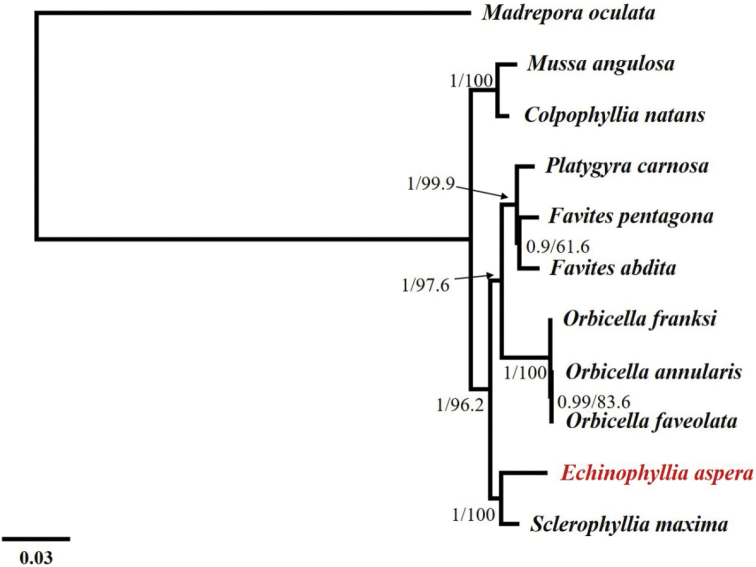
Inferred phylogenetic relationships based on the concatenated nucleotide sequences of 13 mitochondrial protein-coding genes using Bayesian inference (BI) and maximum likelihood (ML). Numbers on branches are Bayesian posterior probabilities and bootstrap percentages.

ML and BI analyses were performed with the concatenated PCG nucleotide data. The topological relationships of two phylogenetic analyses remained consistent, and all analyses provided high support values for all internodes (Figure 7). The phylogenetic tree showed that *E.aspera* clustered most closely with *Sclerophylliamaxima*, which also belongs to Lobophylliidae, but previously was classified as an *Acanthastrea* species (Arrigoni et al. 2015, 2016c). Both species were sister group to Merulinidae, recovering similar relationships with previous studies (Fukami et al. 2008; Kitahara et al. 2010). Indeed, molecular analyses such as the present one, together with traditional studies of micromorphology and microstructure, can help improve modern classification criteria within Scleractinia (Benzoni et al. 2012b; Kitahara et al. 2012, 2016; Budd and Bosellini 2015).

## Conclusion

Limited data are available on the mitogenomes of Lobophylliidae, so the mitochondrial genome of *Echinophylliaaspera* was completed using NGS in the present study. The mitogenome of *E.aspera* was found to be 17,697 bp in length and showed a similar composition in size, low GC content and gene order to mitogenomes already available in Scleractinia. In conclusion, the complete mitogenome of *E.aspera* sequenced and analysed in this study provides essential and important DNA molecular data for further phylogenetic and evolutionary analyses for scleractinian phylogeny.
